# Legume green manure partial replacement of fertilizers enhances soil microbial diversity and sustains wheat yield

**DOI:** 10.3389/fpls.2025.1618555

**Published:** 2025-11-13

**Authors:** Huiyan Wang, Hui Wang, He Yang, Xiaoyu Liu, Xinbao Liu, Yongxiang Guan, Yixin Shen, Jianlong Li, Dianlin Yang, Zhengguo Sun

**Affiliations:** 1 College of Agro-grassland Science, Nanjing Agricultural University, Nanjing, China; 2 Agro-Environmental Protection Institute, Ministry of Agriculture and Rural Affairs, Tianjin, China; 3 Jiangsu Provincial Agricultural Technology Extension Station, Nanjing, China; 4 School of Life Science, Nanjing University, Nanjing, China; 5 Nanjing University (Suzhou) High and New Technology Research Institute, Suzhou, China

**Keywords:** intercropping, green manure, wheat yield, soil properties, bacterial community diversity

## Abstract

**Introduction:**

The excessive use of chemical fertilizers has severely impacted soil quality and the ecological environment of farmland, hindering the development of sustainable agriculture.

**Methods:**

This study evaluated five treatments: conventional monoculture of winter wheat (*Triticum aestivum* L., CK), intercropping of winter wheat with Chinese milk vetch (*Astragalus sinicus* L., IM), crimson clover (*Trifolium incarnatum* L., IC), smooth vetch (*Vicia villosa* Roth., IS), and common vetch (*Vicia sativa* L., IV). Intercropping treatments all resulted in a 30% reduction in chemical fertilizer application compared to monocropping.

**Results:**

The results indicated that the IC treatment yielded wheat grain quantities most similar to CK. When the wheat reaches maturity, the IS treatment resulted in increases in soil pH and soil organic matter (SOM) of 6.73% and 25.76%, respectively (*P* < 0.05). The greatest enhancements of soil urease (URE) and acid phosphatase (ACP) activities were observed with the IM treatment, which showed increases of 32.24% and 57.58%, respectively, compared to wheat monoculture. The IC treatment also enhanced soil sucrose (SUC) activity by 15.24% (*P* < 0.05). Intercropping with leguminous green manure improved the diversity and richness of soil bacterial communities, as evidenced by increases in the Chao 1 and Shannon indices, along with higher relative abundances of Proteobacteria and *Sphingomonas*.

**Discussion:**

Selecting the appropriate leguminous green manure is crucial. Non-climbing varieties are generally preferred. We believe that intercropping crimson clover in wheat fields can not only maintain the yield of the main crop, reduce agricultural production costs, but also improve the micro-ecological environment of the farmland, providing a novel solution for breaking through traditional wheat farming models.

## 1 Introduction

Improving crop production efficiency and maintaining farmland soil quality have become essential strategic initiatives to ensure global food security under the dual challenges of continued global population growth and increasing climate change (Xu et al., 2021). As one of the most important food crops in the world’s food supply system, wheat is widely cultivated and plays a vital role in ensuring basic food rations for the world’s population. As the world’s largest wheat producer and fertilizer user, China has a grain production system that is highly dependent on fertilizer application (Geng et al., 2019; Liu et al., 2017). However, the overuse of chemical fertilizers has not only degraded the soil quality of agricultural lands and further increased agricultural production costs, but also exacerbated agro-ecological pollution (van der Bom et al., 2018; Carvalho, 2017; Chen et al., 2020). Therefore, the research and implementation of scientific and rational fertilization strategies are crucial to promote the sustainable and stable growth of wheat yield, maintain the fertility and health of farmland soils, reduce the negative impact on the natural environment, and promote the sustainable development of agricultural production.

In recent years, legume-based intercropping systems have been widely adopted worldwide. Chinese milk vetch, crimson clover, smooth vetch, and common vetch are some of the more common leguminous green manures used in agricultural production in the middle and lower reaches of the Yangtze River in China. Chinese milk vetch can effectively improve the physicochemical properties of soil, increase the number and diversity of soil microorganisms, and improve soil fertility and crop yield and quality, which is considered to be an important soil nutrition measure (Lin et al., 2011). Crimson clover is a high quality annual fodder grass of the Leguminosae family, which is suitable for winter green manuring in rice fields, but can also be sown as a single crop for pasture establishment and fodder production. Smooth vetch and common vetch are excellent fodder and green manure crops of the wild pea genus of the legume family, rich in protein, calcium, phosphorus and other nutrients, and are also a kind of green manure germplasm resources that are more commonly used in current production. Optimizing soil biodiversity through the additional nitrogen source provided by legume root nitrogen fixation not only effectively increases crop yields (Li et al., 2020; Zhang et al., 2023a), but also enhances soil fertility through interspecific interaction mechanisms (Lange et al., 2015; Spohn et al., 2023). In addition, legumes have the ability to release protons that, when released, can alter the pH of the soil ecosystem, facilitating the mobilization and release of tightly bound organic and inorganic phosphorus sources in the soil into forms that are more readily absorbed and utilized by plants. This mechanism of action not only optimizes soil phosphorus cycling and utilization, but also significantly enhances plant phosphorus uptake in intercropping systems, further promoting healthy plant growth and yield improvement (Li et al., 2007). Interactions between different legume species and their intercropping with wheat vary in terms of crop growth response and soil quality (Zhang et al., 2023b), and it is not clear how crop yields and soil quality will respond to different combinations of legume-wheat intercropping.

Soil microorganisms play a central role in soil nutrient cycling and directly influence soil properties and crop productivity (Van der Heijden et al., 2008; Naylor et al., 2020). It was found that intercropping leguminous green manures increased the diversity and abundance of soil bacterial communities and changed their structure compared to monoculture, thereby optimizing the soil microbial environment. This helps mitigate the negative effects of overfertilization on soil microbes, thus providing more favorable environmental conditions for crop growth (Han et al., 2022; Liu et al., 2021; Gu et al., 2019). The reason for this may be that intercropping increases soil effective nutrients, total nitrogen and soil organic carbon, and increases extracellular enzyme activities (Curtright and Tiemann, 2021; Ma et al., 2022). However, the diversity and composition of soil microbial communities are extremely sensitive to changes in environmental factors (Zhalnina et al., 2015). Thus, intercropping may affect the diversity and composition of soil microbial communities by altering soil nutrient levels and enzyme activities. Most current studies are limited to exploring the effects of intercropping legume green manures with maize and soybean crops on the physicochemical properties and microbial community structure and composition of the cropped soils. In contrast, there is a relative paucity of research on the combination of legume green manures intercropped with wheat, especially on a comprehensive assessment of their overall impact on wheat yield and soil quality by integrating multiple dimensions such as crop productivity, soil quality, and microbial community characteristics.

For many years, we have conducted a series of scientifically rigorous research and experimental efforts centered around utilizing legume green manure as intercrops or in crop rotation systems. Based on this research foundation, we conducted an experiment in the wheat-growing regions of southern Jiangsu, China, to explore the effects of intercropping wheat with legume green manure. We hypothesized that in a wheat system intercropped with leguminous green manure, chemical fertilizer application could be reduced by 30%. Owing to the unique root nodule structure of the leguminous plants, the system could effectively compensate for potential nutrient deficiencies, thereby maintaining wheat yield without decline and simultaneously improving the soil micro-ecological environment. To test this, our objectives were to: (i) investigated the effects of intercropping legume green manures (Chinese milk vetch, crimson clover, smooth vetch, and common vetch) with wheat on soil physicochemical properties and enzyme activities, (ii) assessed the diversity and composition of microbial communities in different intercropping systems, (iii) the effects of intercropping different legume green manures on wheat yield and nutrient uptake and use efficiency. The research outcomes furnish a theoretical groundwork for reducing the utilization of chemical fertilizers and enhancing agricultural production efficiency in winter wheat cultivation.

## 2 Materials and methods

### 2.1 Study site

**Figure 1:**
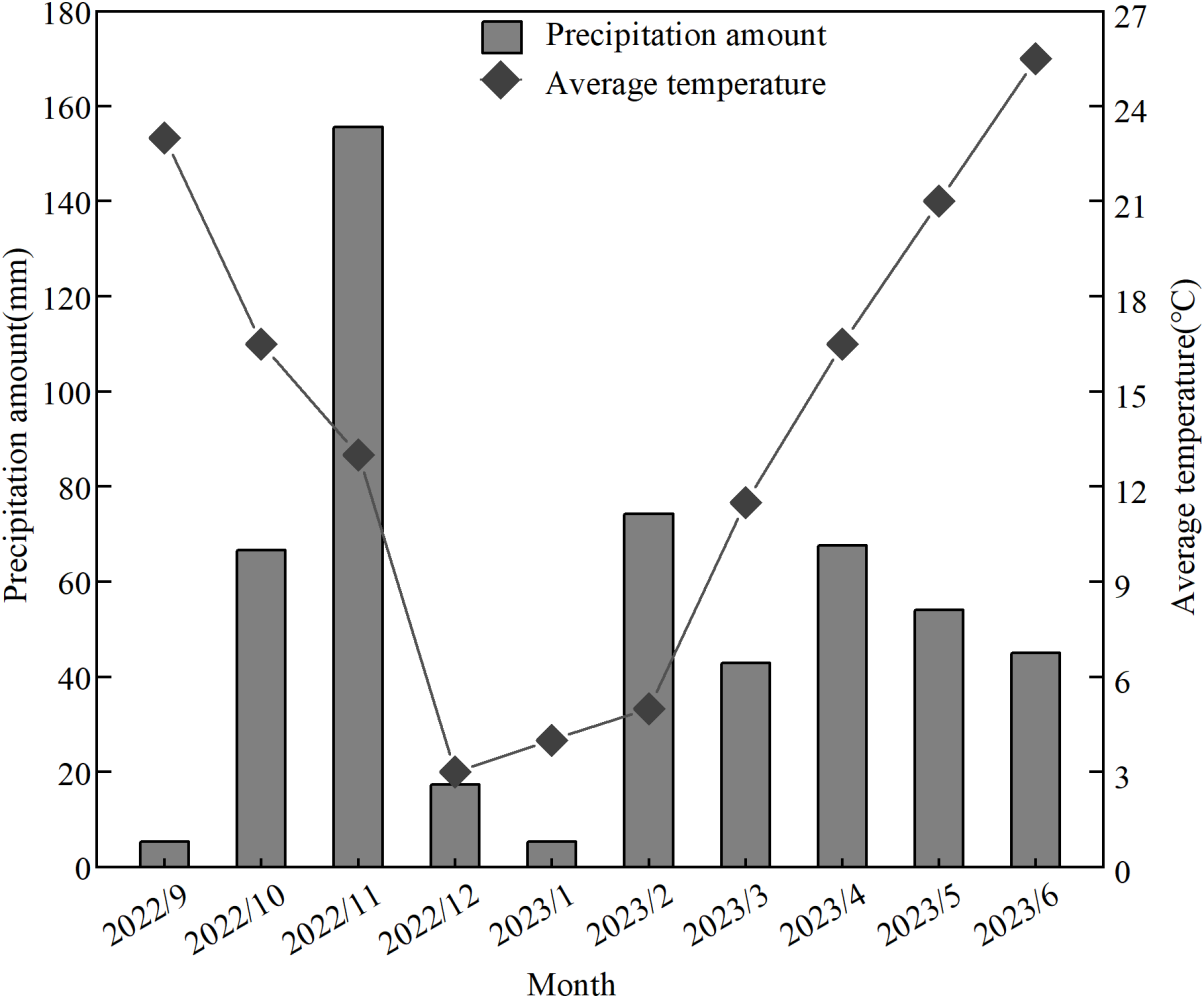
Average monthly temperature and precipitation during the experiment.

The experiment period runs from October 2022 to June 2023. The field experiment was conducted in Huanggang village, Maji town, Luhe District, Nanjing city, Jiangsu Province, China (N 32° 02’ 00”, E 118° 49’ 12”). This area is characterized by a subtropical monsoon climate, featuring high temperatures and heavy rainfall in summer and low temperatures with minimal rainfall in winter. The average annual temperature is 15.1°C, with a total precipitation of 979.5 mm and a frost-free period of 226 days. The average monthly temperature and precipitation during the experiment are shown in **Figure 1**. The soil type at the experimental site is yellow–brown soil. The basic physical and chemical properties of the 0–20 cm soil layer are as follows: pH, 5.76 ± 0.02; soil organic matter (SOM), 10.78 ± 0.10 g kg^−1^; total nitrogen, 0.59 ± 0.01 g kg^−1^; total phosphorus, 0.37 ± 0.02 g kg^−1^; total potassium, 8.36 ± 0.12 g kg^−1^; alkali-hydrolyzed nitrogen (AN), 105.42 ± 0.78 mg kg^−1^; and rapidly AP, 10.39 ± 0.41 mg kg^−1^.

### 2.2 Experimental design and sampling

**Figure 2:**
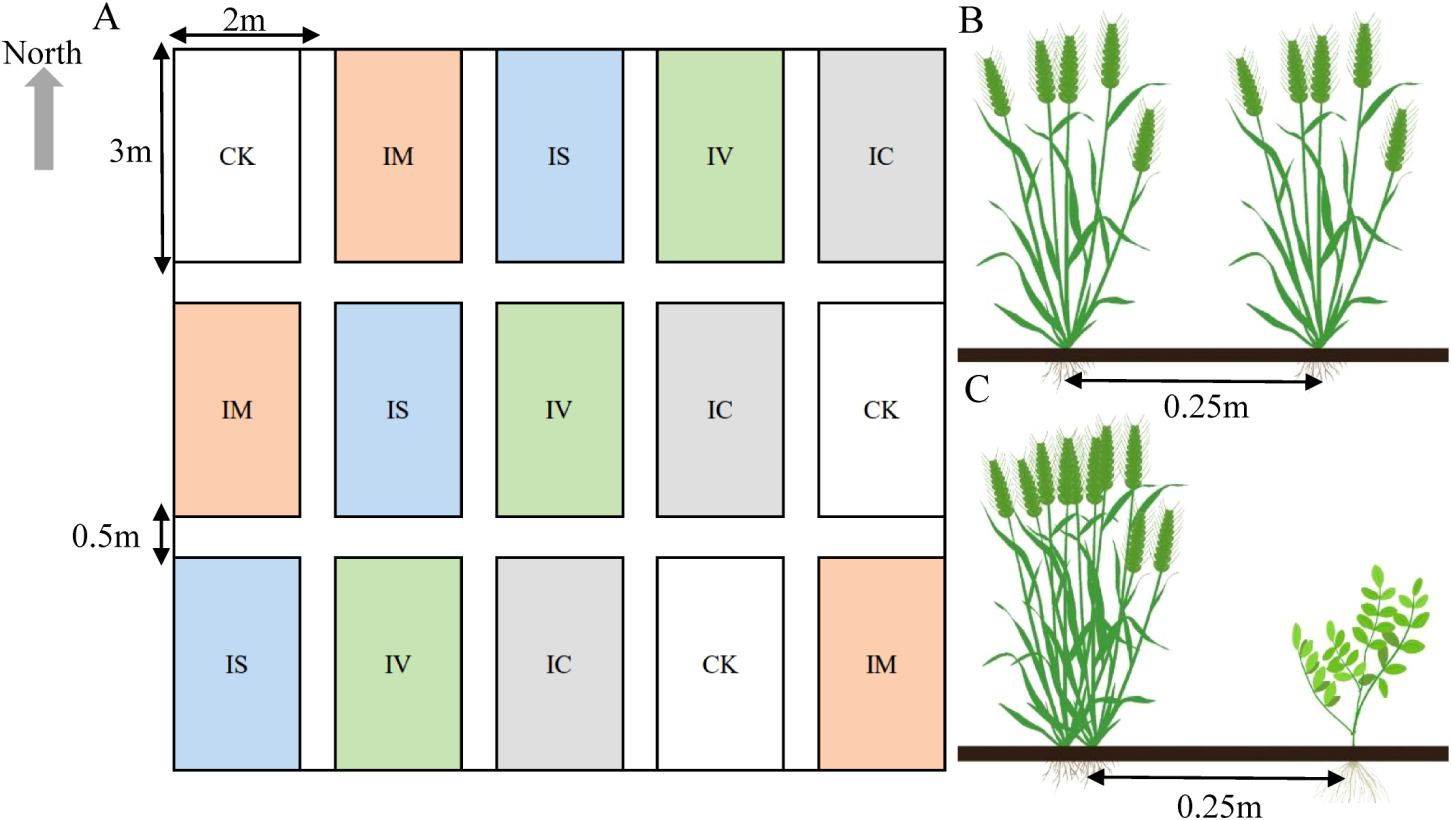
A random block design was adopted in the field trial, and the distribution of each cell is shown in **(A)**. **(B)** shows the planting methods used for wheat in the monoculture wheat plot. **(C)** shows the planting of wheat in an intercropping legume green manure plot. CK stands for the wheat monocropping treatment, IM stands for the wheat intercropped with Chinese milk vetch treatment, IC stands for the wheat intercropped with clover treatment, IS stands for the wheat intercropped with smooth vetch treatment, and IV stands for the wheat intercropped with common vetch treatment.

The community experiment consisted of five treatments: conventional monoculture of winter wheat (*Triticum aestivum* L., CK), intercropping of winter wheat with Chinese milk vetch (*Astragalus sinicus* L., IM), intercropping with crimson clover (*Trifolium incarnatum* L., IC), intercropping with smooth vetch (*Vicia villosa* Roth., IS), and intercropping with common vetch (*Vicia sativa* L., IV). Each treatment was replicated three times, resulting in a total of 15 communities. Each community covered an area of 6 m² (2 m × 3 m) and was separated by 50 cm wide and 30 cm high drainage ditches. The wheat was sown on October 20, 2022. In the monoculture wheat treatment, the sowing density was 2.7 × 10^6^ plants per hectare with a row spacing of 25 cm. The intercropping of leguminous green manure was implemented by alternating one row of wheat with one row of green manure, with a row spacing of 25 cm. The sowing density for each wheat row was twice that of the monoculture wheat plot. The sowing densities for Chinese milk vetch, crimson clover, smooth vetch, and common vetch were each 1.005 × 10^6^ plants/hm². The sowing densities for both monoculture wheat and intercropped leguminous green manure were standardized. The wheat was planted in an east-west direction. In the monoculture wheat plot, 12 rows of wheat were planted. In the intercropping green manure plot, wheat and green manure were planted in alternating rows, resulting in a total of 6 rows of wheat and 6 rows of green manure. The distribution and planting methods for each plot are illustrated in **Figure 2**.

During the growth period of monoculture wheat, 270 kg of pure nitrogen (N), 105 kg of phosphorus pentoxide (P_2_O_5_), and 150 kg of potassium oxide (K_2_O) were applied per hectare. In the wheat intercropping system, the application of leguminous green manure was reduced by 30% compared with that of monoculture wheat, resulting in the application of 189 kg of pure N, 73.5 kg of P_2_O_5_, and 105 kg of K_2_O per hectare. The fertilizers used included a compound fertilizer (N_2_:P_2_O_5_:K_2_O = 18:18:8), urea (containing 46.4% N), and potassium sulfate (containing 52% K_2_O). All the compound and potassium fertilizers were applied as base fertilizers, with 60% of the nitrogen fertilizer applied at planting and 40% applied as topdressing after the wheat booting stage. Irrigation was conducted as necessary on the basis of soil moisture to manage diseases, pests, and weeds.

Soil samples were collected randomly from the topsoil (0–20 cm) using the S-shaped sampling method during the overwintering (S1), jointing (S2), heading (S3), and ripening (S4) stages of wheat, with each stage sampled three times. A portion of each sample was sieved through a 2 mm sieve to evaluate the soil physicochemical properties, whereas another portion was stored in a -80°C freezer for microbial community analysis. One meter of uniformly growing wheat plants was randomly selected from each community for yield testing. Additionally, ten uniformly growing wheat plants were randomly chosen to assess agronomic traits and nutrient absorption and utilization indicators.

### 2.3 Determination of experimental indicators

#### 2.3.1 Determination of yield, agronomic traits and nutrient accumulation in wheat

During the maturation stage of wheat, uniformly growing plants from each plot were manually harvested at a depth of 1 m to evaluate yield and grain moisture content. The final yield was standardized to a moisture content of 13%, and various agronomic traits were recorded. The key traits included spike number, grains per spike, thousand-grain weight, and stem count. To determine the number of grains per ear, ten uniformly growing wheat ears were randomly selected from each plot, and the average grain count was calculated. The thousand-grain weight refers to the weight of 1,000 grains. Wheat grains and dried straw samples were ground using a grinder (MM400, RETSCH, Germany). The total nitrogen content was then determined via the Kjeldahl method with 5% boric acid and 40% NaOH (Chapman et al., 1961). The total phosphorus content is estimated via spectrophotometry (Watanabe and Olsen, 1965), and the total potassium content is measured with a flame photometer (Chapman et al., 1961). Nutrient absorption levels (N, P, K) in each organ were calculated by multiplying the dry weight (kg ha^−1^) by the corresponding nutrient concentration (g kg^−1^). Total nutrient absorption in the aboveground biomass (kg ha^−1^) is determined by summing nutrient absorption across all organs.

#### 2.3.2 Determination of soil physicochemical properties

The soil moisture content (SMC) was determined via the drying method (Chehab et al., 2020). The soil bulk density (SBD) was measured via the core method (Wang et al., 2021). The soil porosity (SP) was calculated from the SBD and density. The pH value was measured via the potentiometric method with a soil-to-water ratio of 1:2.5 (Li H. et al., 2021). The alkaline hydrolysis nitrogen content was measured via the alkaline hydrolysis diffusion method. AP was determined via the sodium bicarbonate extraction colorimetric method (Xiong et al., 2015), and SOM was measured via the potassium dichromate volumetric method with external heating (Wang et al., 2016). The soil enzyme activity was determined following methods from Zhao et al. (2016). Sucrase (SUC) was measured via the 3,5-dinitrosalicylic acid colorimetric method. Urease (URE) was measured via the sodium phenolate–sodium hypochlorite colorimetric method. Acid phosphatase (ACP) activity was assessed via the disodium phenyl phosphate colorimetric method. CAT activity was measured via the permanganate titration method.

#### 2.3.3 Determination of soil bacterial communities

The soil samples stored at -80°C were retrieved, and 0.5 g was weighed for microbial DNA extraction via the Omega Biotek e.z.n.a.^®^ reagent kit (Norcross, GA, USA). The V3-V4 variable region of the bacterial 16S rRNA gene, approximately 468 bp long, was selected for sequencing. The amplification primers used were 338F (5’-block+ACTCCTACGGGAGGCAGCA-3’) and 806R (5’-GACTACHVGGGTWTCTAAT-3’).

The bacterial PCR conditions included initial denaturation at 95°C for 5 minutes. This was followed by 30 seconds of denaturation at 95°C, 30 seconds of annealing at 55°C, and 45 seconds of extension at 72°C. This cycle was repeated 27–30 times, with a final extension at 72°C for 5 minutes. A 6-base barcode was added to each sample for identification. The PCR was performed in a 30 μL mixture, which consisted of 15 μL of high-fidelity DNA polymerase, 1 μL each of forward and reverse primers (10 μM), and 20 ng of template DNA.

The amplicon was extracted from a 2% agarose gel and purified using the AxyPrep DNA Gel Extraction Kit (Axygen Biosciences, Union City, CA, USA) according to the manufacturer’s instructions. It was then quantified with a Qubit^®^ 3.0 (Life Technologies, Invitrogen). The amplicons were mixed with an average of 20 different barcodes and constructed into an Illumina end-to-end library via Illumina’s genomic DNA library preparation procedure. Finally, the amplicon library was subjected to paired-end sequencing according to the standard protocol on the Illumina NovaSeq 6000 platform.

### 2.4 Statistical analysis

The data in this study were organized via Microsoft Excel and analyzed for normality and variance via SPSS version 25.0 (IBM, USA). One-way analysis of variance (ANOVA) and Duncan’s honestly significant difference (HSD) test (*P* < 0.05) were employed to assess differences in soil physicochemical properties, enzyme activity, wheat yield, and constituent factors across various intercropping treatments.

The original microbiome sequences were processed via QIIME 2 (2019) software. This included quality filtering, merging, and optimizing the raw sequencing data. For the 16S rRNA gene analysis, the default reference was the Silva 138 rRNA database (Edgar, 2013). Species classification information corresponding to each amplicon sequence variant (ASV) was obtained by aligning ASV sequences with the Silva bacterial database.

QIIME 2 software was used to generate rarefaction curves, which helped evaluate whether the current sample size accurately reflected changes in the bacterial community structure. The diversity indices of the soil bacterial communities were calculated, with a focus on alpha diversity through the Chao1 and Shannon indices. These indices indicate the richness and diversity of bacterial communities, respectively. Nonmetric multidimensional scaling (NMDS) analysis was performed via the vegan package in R, whereas statistical tests were conducted via permutation multivariate analysis of variance (PERMANOVA). Additionally, redundancy analysis (RDA) was used to demonstrate the associations between the soil bacterial community composition and physicochemical factors.

## 3 Results

### 3.1 Wheat yield, agronomic traits, and nutrient accumulation and distribution

#### 3.1.1 Wheat yield and agronomic traits

The effects of intercropping various legume green manures on wheat yield and agronomic traits are summarized in **Table 1**. There were no significant differences in wheat yield between the intercropping treatments (IM and IC) and monocropped wheat (*P* ≥ 0.05). However, yields in the control under the IS and IV treatments significantly decreased (*P* < 0.05), with reductions of 7.51% and 5.98%, respectively. Compared with those of monocropped wheat, the number of grains per spike and the weight of 1,000 grains significantly increased in the IM, IC, and IS treatments (*P* < 0.05). The IM treatment resulted in the greatest increase in the number of grains per spike, which increased by 18.63% compared with the CK. The largest increase in the 1000-grain weight was observed in the IC treatment, which was 8.04% higher than that of the CK. Conversely, these intercropping treatments resulted in a significant reduction in the number of tillers and panicles compared with those of the monocropped wheat (*P* < 0.05). Among the treatments, the IV treatment showed the most significant decrease in both the number of wheat stem nodules and the number of grains per spike, with a decrease of 13.75% in the former and 29.41% in the latter.

**Table 1:** Effects of intercropping with different types of legume green manure on wheat agronomic traits.

Treatments	Tillers number (10^4^ hm^-2^)	Spike number (10^4^ hm^-2^)	Kernel number	1000- Kernel mass (g)	Yield (kg hm^-2^)
CK	1101.53 ± 26.87a	362.56 ± 10.25a	37.41 ± 1.53a	44.78 ± 0.10c	5310.28 ± 63.55a
IM	1004.27 ± 41.09bc	286.20 ± 3.89b	44.38 ± 1.52b	46.57 ± 0.05b	5180.88 ± 135.57a
IC	1062.55 ± 35.59b	295.69 ± 1.56b	42.04 ± 0.99b	48.38 ± 0.03a	5248.86 ± 96.36a
IS	986.33 ± 43.24c	282.39 ± 14.38b	42.73 ± 1.53b	46.83 ± 0.84b	4939.15 ± 21.09b
IV	968.40 ± 26.90c	280.17 ± 3.68b	43.09 ± 0.94b	47.43 ± 1.02ab	5010.50 ± 67.43b

CK stands for the wheat monocropping treatment, IM stands for the wheat intercropped with Chinese milk vetch treatment, IC stands for the wheat intercropped with clover treatment, IS stands for the wheat intercropped with smooth vetch treatment, and IV stands for the wheat intercropped with common vetch treatment. Different lowercase letters (a, b, c) in the same growth period indicate significant differences between treatments (*P* < 0.05).

#### 3.1.2 Accumulation of nutrients in the aboveground parts of wheat

**Figure 3:**
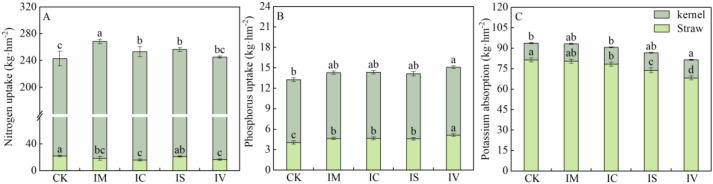
Effects of intercropping with different types of legume green manure on the above-ground nutrient accumulation of wheat. Nitrogen accumulation in kernels and straw **(A)**, phosphorus accumulation in grains and straw **(B)**, and potassium accumulation in grains and straw **(C)**. Different lowercase letters (a, b, c) indicate significant differences between treatments (*P* < 0.05).

The effects of intercropping various leguminous green manures on the accumulation of nitrogen, phosphorus, and potassium in the aboveground parts of wheat at maturity are illustrated in **Figure 3**. Compared with the monocropping wheat, both the IM and IC treatments significantly increased total nitrogen accumulation in the aboveground parts (*P* < 0.05) for nitrogen and phosphorus accumulation (2A and 2B). Notably, the IM treatment resulted in the greatest total nitrogen and potassium accumulation in the aboveground parts. Intercropping with different leguminous green manures increased nitrogen and phosphorus accumulation in wheat grains but reduced nitrogen levels in wheat stems. This finding indicates that intercropping with leguminous green manures shifts nitrogen allocation from the stems to the grains. Compared with monocropping wheat, intercropping with various legume green manures resulted in decreased total potassium in the aboveground parts of wheat, less potassium flow to the grains, and increased potassium flow to the stems.

#### 3.1.3 Accumulation and utilization efficiency of nutrients in the aboveground parts of wheat

**Table 2** presents the effects of intercropping various leguminous green manures on the absorption and utilization efficiency of nitrogen, phosphorus, and potassium in the aboveground parts of wheat. Compared with those of monoculture wheat, the absorption efficiencies of nitrogen, phosphorus, and potassium in the IM, IC, IS, and IV treatments were significantly greater (*P* < 0.05). The increases ranged from 41.11% to 57.78% for nitrogen, 46.15% to 69.23% for phosphorus, and 25.81% to 43.55% for potassium. However, the nitrogen and phosphorus utilization efficiencies in the IM, IC, IS, and IV treatments decreased relative to those in the CK (*P* < 0.05), with reductions of 5.50% to 13.60% for nitrogen and 9.58% to 20.76% for phosphorus. On the other hand, potassium utilization efficiency increased overall in all the treatments compared with that in the CK, with the IV treatment achieving the most significant improvement at 8.47%. Based on these results, intercropping with leguminous green manure crops enhances nitrogen and phosphorus absorption efficiency but reduces their utilization efficiency.

**Table 2:** Effects of intercropping different legume green manures on aboveground nutrient uptake and utilization efficiency in wheat.

Treatments	NUPE (kg/kg)	PUPE (kg/kg)	KUPE (kg/kg)	NUTE (kg/kg)	PUTE (kg/kg)	KUTE (kg/kg)
CK	0.90 ± 0.04d	0.13 ± 0.02b	0.62 ± 0.01d	21.88 ± 0.75a	400.77 ± 20.04a	56.66 ± 0.98bc
IM	1.42 ± 0.03a	0.22 ± 0.02a	0.89 ± 0.06a	19.30 ± 0.45c	362.37 ± 6.46b	55.56 ± 1.01c
IC	1.34 ± 0.05bc	0.20 ± 0.02a	0.86 ± 0.02ab	20.74 ± 0.57b	365.73 ± 12.50b	57.87 ± 0.07b
IS	1.36 ± 0.01b	0.19 ± 0.04a	0.83 ± 0.01bc	19.26 ± 0.13c	349.93 ± 12.84bc	57.00 ± 0.78bc
IV	1.27 ± 0.04c	0.21 ± 0.03a	0.78 ± 0.02c	20.44 ± 0.29b	331.86 ± 8.49c	61.46 ± 0.97a

NUPE stands for nitrogen uptake efficiency, PUPE stands for phosphorus uptake efficiency, and KUPE stands for potassium uptake efficiency. NUTE stands for nitrogen utilization efficiency, PUTE stands for phosphorus utilization efficiency, and KUPE stands for potassium utilization efficiency. Different lowercase letters (a, b, c, d) indicate significant differences between treatments (*P* < 0.05).

### 3.2 Physical and chemical properties of the soil

#### 3.2.1 Soil nutrient content

**Figure 4:**
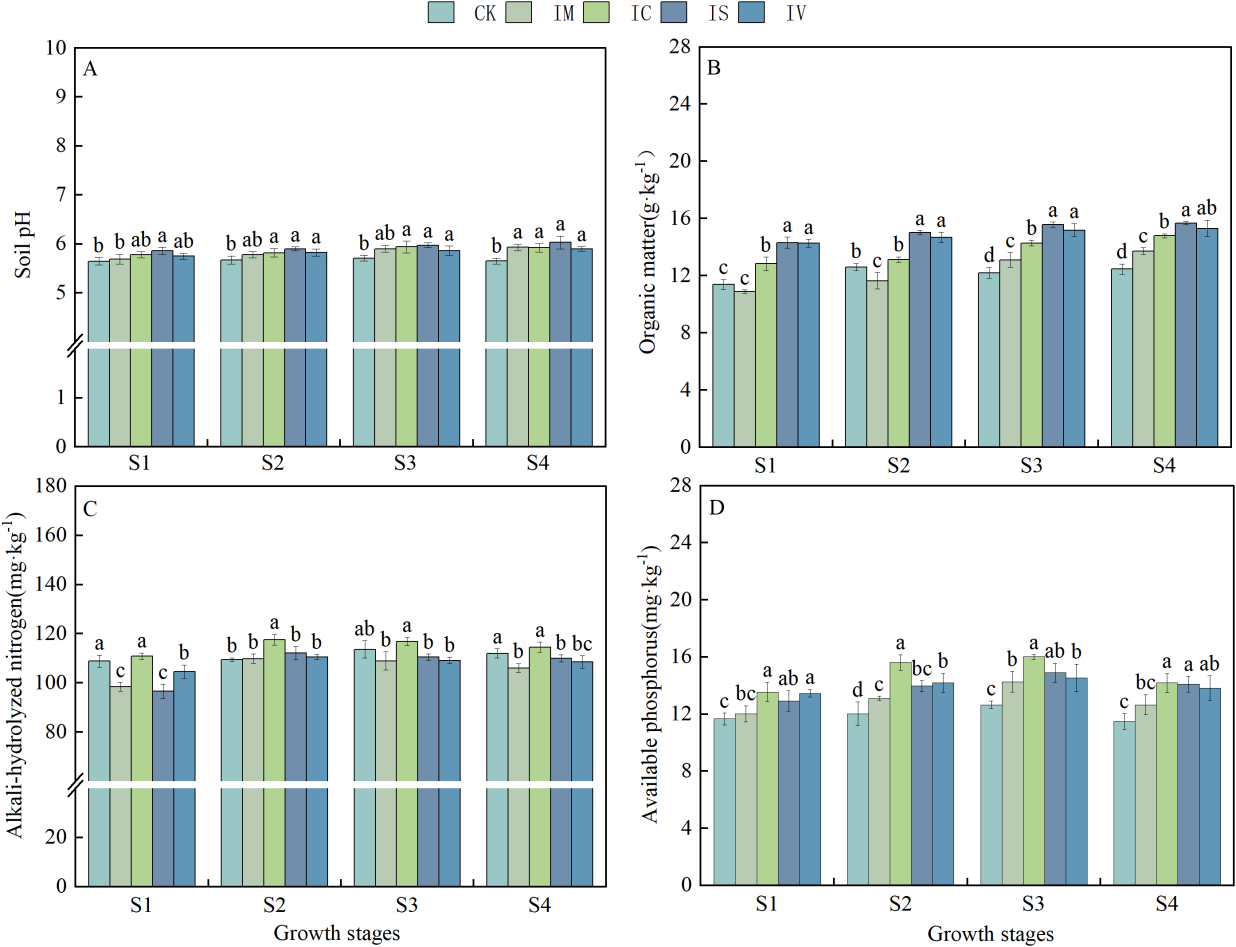
Effects of intercropping with different legume green manures on the soil nutrient content. Effects of intercropping various legume green fertilizers on soil pH **(A)**, organic matter **(B)**, alkali-hydrolyzed nitrogen **(C)**, and available phosphorus **(D)**. Different lowercase letters (a, b, c, d) indicate significant differences between treatments (*P* < 0.05). S1 represents the overwintering stage, S2 represents the jointing stage, S3 represents the heading stage, and S4 represents the ripening stage.

As the reproductive period progressed, both the soil pH and the SOM content of the intercropped leguminous green manure treatments exhibited an increasing trend compared to the CK (**Figures 4A, B**). The overwintering stage (S1), jointing stage (S2), heading stage (S3), and ripening stage (S4) all exhibited significant improvements in soil pH as a result of the IS treatment, which increased by 3.71%, 4.06%, 4.55%, and 6.73%, respectively, compared to the CK. During S1, S2, S3, and S4, SOM was significantly higher (*P* < 0.05) in the IS and IV treatments compared to the CK treatment. The most substantial increase in SOM content was observed in the IS treatment across all periods, with increases of 25.53%, 19.13%, 27.62%, and 25.76%, respectively, compared to CK during all fertility periods. The AN content varied significantly across different periods (**Figure 4C**). In the S2 period, the soil AN content in the IM, IC, IS, and IV treatment test plots exhibited an increasing trend compared to the S1 period, with increases of 11.59%, 6.04%, 15.98%, and 5.65%, respectively. At this time, the IS treatment test plot exhibited the largest increase in AN content, which was 15.44 mg kg^-1^ higher than during the S1 period. In contrast, the IV treatment test plot showed the smallest increase in AN content, with an increase of only 5.91 mg kg^-1^ compared to the S1 period. During the S3 and S4 periods, the content of AN gradually decreased. There were significant differences in the quick-acting phosphorus content among the various treatments; however, the overall trend indicated that the AP content for each treatment initially increased and then decreased as the reproductive period progressed (**Figure 4D**). During S2 and S3, the AP content in the IM, IC, IS, and IV treatments gradually increased compared to S1. The most significant increase was observed in the IM treatment at S3, which was 2.24 mg kg^−1^ higher than that of S1. During the S4 period, the impact of intercropping leguminous green manure treatments on the AP content in wheat exhibited a decreasing trend compared to the S3 period.

#### 3.2.2 Soil enzyme activities

**Figure 5:**
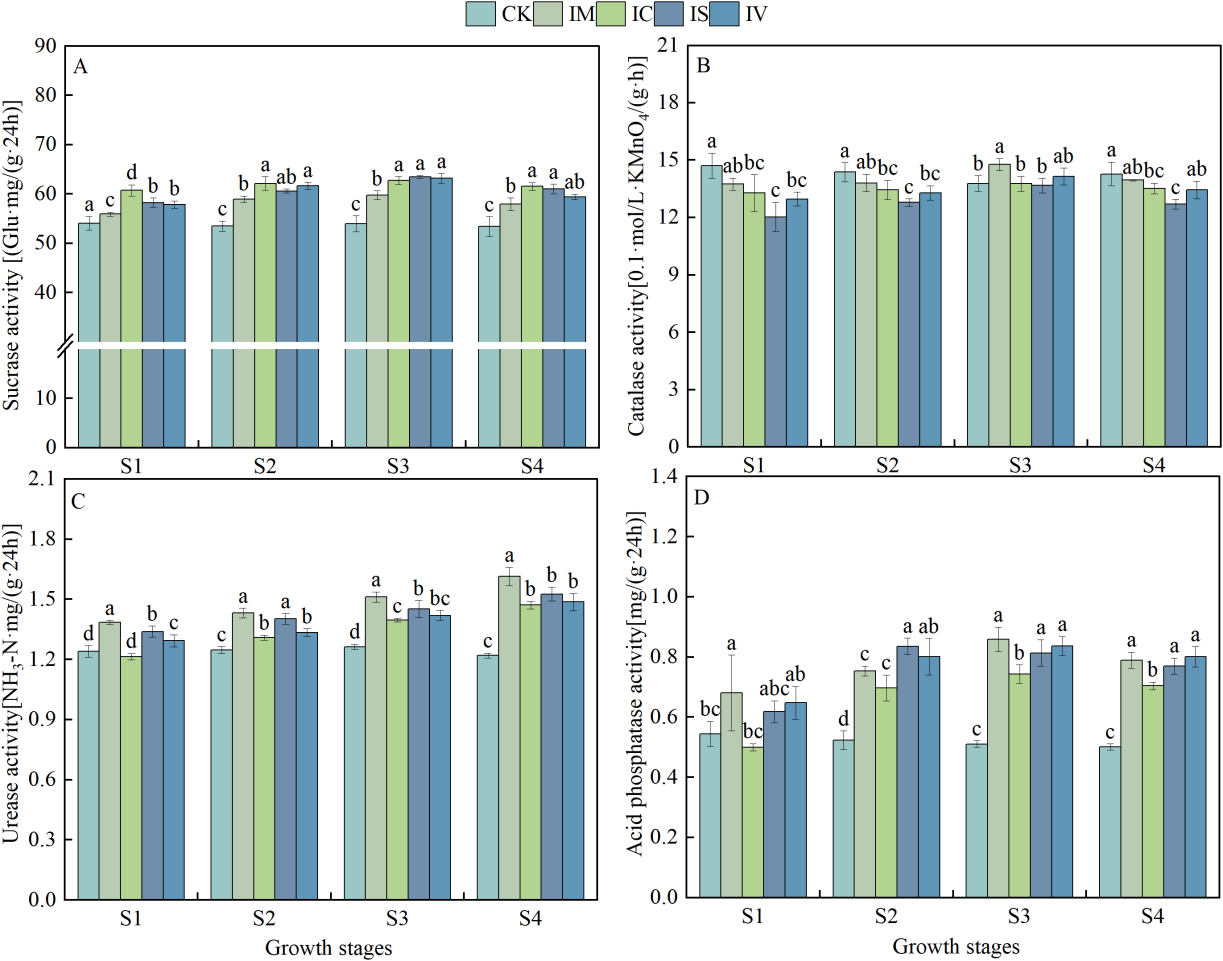
Effects of intercropping with different types of legume green manure on soil enzyme activity. Effects of intercropping various legume green manures on sucrase **(A)**, catalase **(B)**, urease **(C)**, and acid phosphatase **(D)** activity. Different lowercase letters (a, b, c) indicate significant differences between treatments (*P* < 0.05).

With advancements in fertility processes, the SUC and CAT activities of each intercropped leguminous green manure treatment exhibited a trend of initially increasing and then decreasing. (**Figures 5A, B**) In contrast, there was no significant change in soil SUC activity in the CK test plot. Soil SUC activity was significantly higher (*P* < 0.05) in the IM, IC, IS, and IV treatments compared to the CK during S1, S2, S3, and S4. The CAT activity of all intercropped leguminous green manure treatments exhibited a gradual increase during the S1, S2, and S3 periods. In the S3 period, the soil CAT activity for the IM, IC, IS, and IV treatments increased by 7.42%, 3.71%, 13.61%, and 9.14%, respectively, compared to the S1 period. During the S4 period, the CAT activity of all intercropped leguminous green manure treatments exhibited a gradual decline compared to the S3 period. Specifically, the soil CAT activity for the IM, IC, IS, and IV treatments decreased by 2.30%, 5.63%, 12.48%, and 6.24%, respectively, in comparison to the CK. The URE activity of each intercropped legume green manure treatment exhibited a gradual increase as the fertility process progressed. (**Figure 5C**) During different fertility periods, all treatments demonstrated the most significant enhancement of soil URE activity through IC treatment. Soil urease activity increased by 32.24%, 20.61%, 25.09%, and 21.84%, respectively, compared to the CK during the fourth sampling period S4. The trends in soil ACP activity, as well as soil SUC and CAT activity, during the primary fertility period of each intercropped legume green manure treatment were notably consistent (**Figure 5D**). These trends exhibited an initial increase followed by a subsequent decrease. Specifically, the activities gradually rose, reaching their maximum values during the S1, S2, and S3 periods, before beginning to decline in the S4 period.

### 3.3 Soil bacterial community composition and diversity

#### 3.3.1 Composition of the soil bacterial community structure

**Figure 6:**
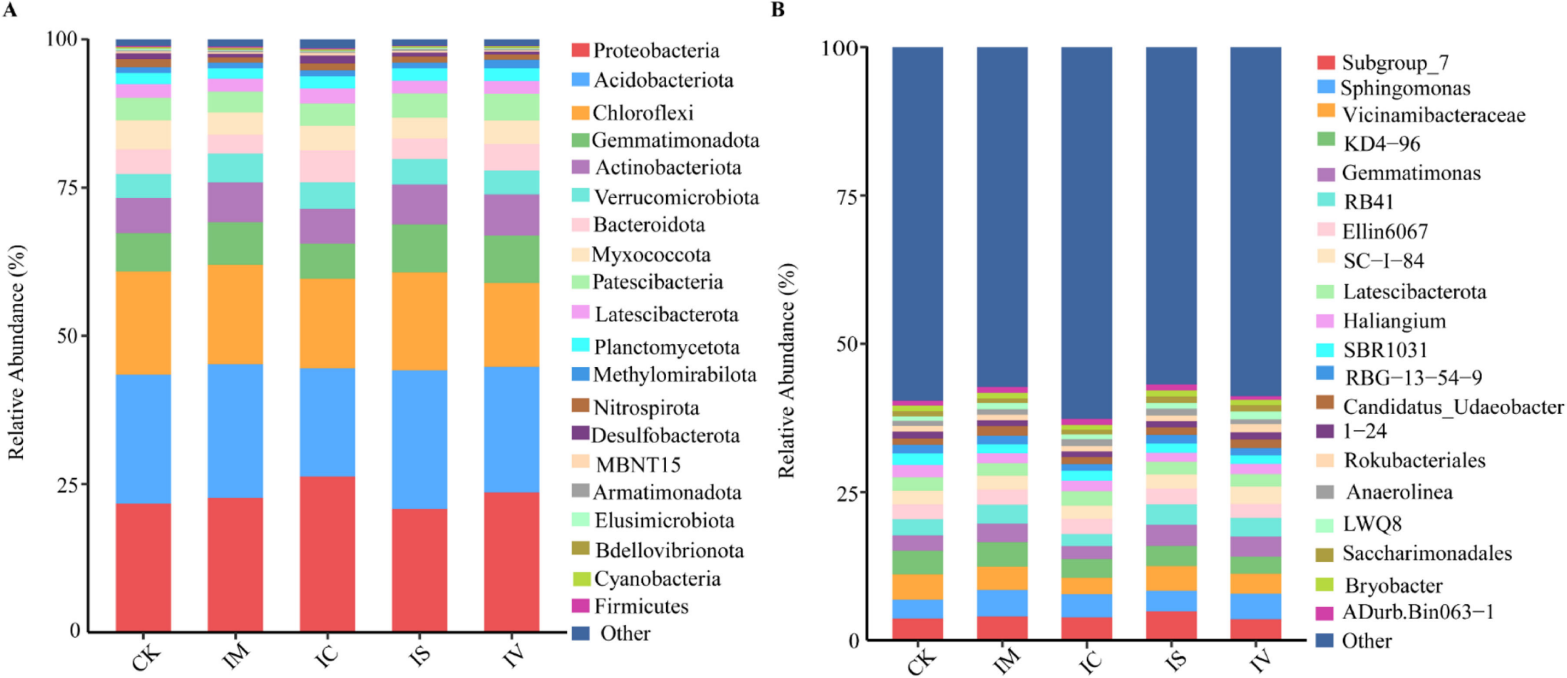
Analysis of the differences in soil bacterial community composition when intercropped with various legume green manures. **(A)** shows the relative abundance of soil bacterial communities at the phylum classification level, and **(B)** shows the relative abundance of soil bacterial communities at the genus classification level.

The effects of intercropping various leguminous green manures on the composition of the soil bacterial community structure are illustrated in **Figure 6**. Among the leguminous green manure treatments, Proteobacteria had the highest relative abundance (6A), followed by Acidobacteriota, Chloroflexi, and Gemmatimonadota, which contributed 21.75–26.32%, 18.24–23.37%, 14.15–17.38%, and 5.97–8.10% of the total bacterial count, respectively. Compared with monoculture wheat, bacterial taxa with a relative abundance greater than 1% in the soil community for the IM, IC, IS, and IV treatments represented 95.17%, 97.29%, 96.10%, 96.59%, and 96.70% of the total bacteria, respectively. Compared with those in monoculture wheat, the relative abundances of Zygomycota, Actinobacteria, and Proteobacteria in the IM and IV treatments increased, whereas the relative abundance of Chloroflexia decreased across all intercropping treatments. These findings indicate that intercropping different leguminous green manures elicits diverse responses in the predominant soil bacteria. At the taxonomic level of the soil bacterial community composition (6B), the IM, IC, and IS treatments increased the relative abundances of the *Subgroup_7*, *Ellin6067*, and *Sphingomonas* genera compared with those in the CK. Overall, the relative abundance of the *Subgroup_7* genus in the soil treated with IS was the highest, increasing by 33.21% compared with that in the CK, whereas the *Sphingomonas* genus presented the greatest relative abundance in the IM treatment, with a 40.09% increase compared with that in the CK. In contrast, intercropping with different leguminous green manures resulted in a reduction in the relative abundance of the *Vicinamibacteraceae* and *Haliangium* genera.

#### 3.3.2 Diversity of soil bacterial communities

**Figure 7:**
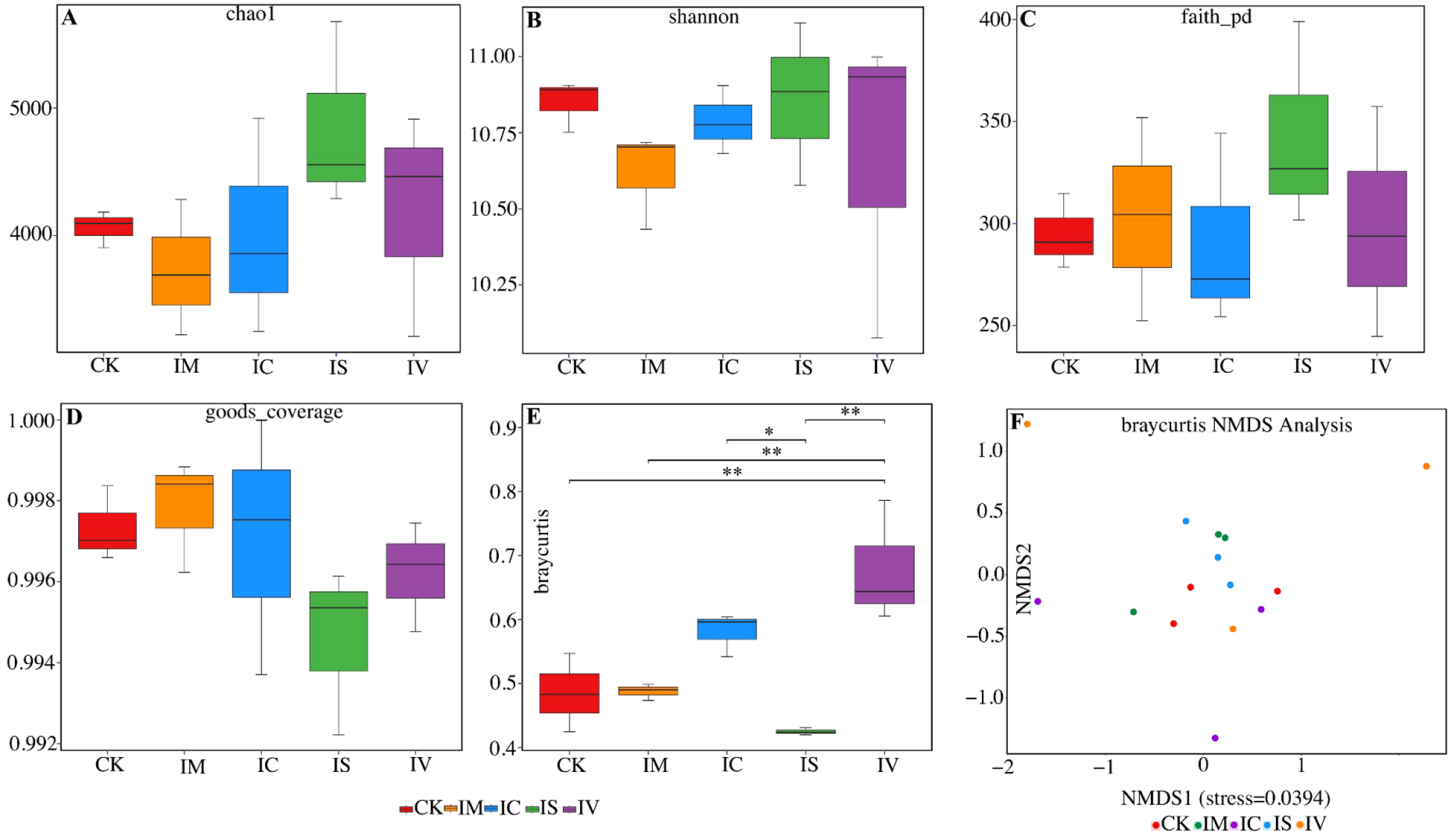
Analysis of the differences in the diversity of the soil bacterial community in response to intercropping with different legume green manures. Effects of intercropping different legume green fertilizers on soil bacterial communities: Chao1 index **(A)**, Shannon index **(B)**, Faith_pd index **(C)**, Good’s coverage **(D)**, substitution multiple variance analysis **(E)**, and NMDS analysis **(F)**. *indicates a significant difference, while the symbol, **indicates an extremely significant difference.

The effects of intercropping various leguminous green manures on soil bacterial community diversity are illustrated in **Figure 7**. The average Chao1 index values, ranked from highest to lowest, are as follows: IS, IV, IC, CK, and IM (7A). These findings indicate that intercropping wheat with Chinese milk vetch does not increase soil bacterial richness. In contrast, intercropping with crimson clover, smooth vetch, or common vetch increased soil bacterial richness. The Shannon indices, arranged from high to low, were as follows: IS, IV, CK, IC, and IM (7B), demonstrating that the IS and IV treatments improved community diversity. The Faith_pd index revealed that different leguminous green manure treatments increased the genetic diversity of the soil bacterial communities, with the IS treatment having the most significant positive effect (7C).

The Good’s coverage index for each treatment ranged from 0.992 to 1, indicating adequate species coverage and sufficient sampling for accurately representing changes in bacterial community diversity (7D). The stress value from the NMDS results was 0.0394, which is below the threshold of 0.2 (7F). This suggests minimal differences in bacterial communities among the treatments. The proximity observed between the CK treatment and the IM, IC, and IS treatments indicates that these intercropping leguminous green manures led to only slight differences in soil bacteria compared with those in the CK. Furthermore, the PERMANOVA intergroup difference analysis (7E) of wheat monocultures versus various intercropped leguminous green manures supported the NMDS findings, confirming significant differences in the beta diversity of bacterial communities across the treatments. These findings confirm that intercropping with different leguminous green manures distinctly affects the composition of the soil bacterial community structure.

## 4 Discussion

### 4.1 Effects of intercropping with different leguminous cover crops on wheat yield, agronomic traits, and nutrient accumulation and distribution

In the present study, intercropping of different legume green manure treatments significantly increased the number of grains and thousand kernel weight of wheat spikes while decreasing the number of spikes and tillers compared to monoculture wheat. This may be due to the good long-term synergistic effect between ecological factors such as water, fertilizer, gas, light, temperature, etc. in the near-surface layer under intercropping cultivation, where crops are able to utilize natural conditions such as land resources, light, water and nutrients more effectively. It was also found that the intercropping legume green manure kept the sabre leaves of the wheat bright green during the later stages of growth, thus providing sustained productivity for continued photosynthesis and facilitating the transfer of more nutrients to the wheat grain, which in turn increased the number of spikes and thousand kernel weight of the wheat (Nasar et al., 2022). We increased the seeding density of wheat per row in the intercropping pattern, and too high seeding density leads to ineffective tillering, more pollen abortion, wasted nutrient and water use, and shading that leads to premature plant senescence (Jiang et al., 2023), which affects tillering and spike formation in wheat. In addition, wheat yields of both the intercropped smooth vetch and intercropped common vetch treatments were reduced compared to the wheat monoculture, probably because both legume green manures are climbers (Li et al., 2021), which would entangle on the wheat stalks during the flowering period, interfering with wheat irrigation and ultimately leading to reduced yields. Wheat intercropped with Chinese milk vetch and wheat intercropped with crimson clover treatments were essentially the same in terms of yield compared to wheat monoculture, suggesting that wheat is not affected by stem entanglement of these two legume green manures in the later stages of growth (Bista and Dahal, 2018). Competition between plants became more intense compared to the control, which weakened their ability to regenerate tillers and led to fragmentation of nutrient distribution. At the same time, pollen abortion increased and grain yield and thousand kernel weight decreased.

In intercropping systems, different crop combinations have complementary effects on nutrient absorption and utilization in terms of time and space (Zhang and Li, 2003). Compared with monocultures, intercropping enhances nutrient absorption capacity, thereby increasing yield (Zhang et al., 2015). In our study, the IM, IC, IS, and IV treatments significantly increased aboveground nitrogen accumulation (*P* < 0.05) relative to that of monoculture wheat. This increase may result from the strong nitrogen fixation ability of leguminous green manure, which increases the nutrient pool in the soil and increases nitrogen availability. This, in turn, increases the nitrogen content and uptake in intercropped grass crops (Dos Santos Sousa et al., 2022). Research has indicated that intercropping corn with alfalfa can increase nitrogen accumulation in the aboveground parts of corn and improve its nitrogen absorption and utilization (Shao et al., 2020). Additionally, Van Deynze et al. (2018) reported that, in intercropping systems, leguminous crops transfer nitrogen to gramineous crops through various mechanisms, accounting for approximately 20% to 82% of total nitrogen uptake by those crops. This finding aligns with our results.

Leguminous green manure provides a direct nitrogen source for crops and significantly enhances soil nitrogen utilization efficiency and retention (Ashraf et al., 2004). This occurs through two key mechanisms: it increases nitrogen absorption directly and increases both AN and AP levels in the soil (Gao et al., 2024). This study revealed that, compared with monoculture wheat, intercropping with leguminous green manure reduced the soil AN content. As a result, it led to decreased nitrogen and phosphorus utilization efficiency in wheat. Research on maize–soybean intercropping has shown that such practices increase nitrogen content, uptake, and use efficiency in maize, especially with optimized nitrogen fertilizer applications. This improvement is largely due to better rhizosphere conditions and nutrient contributions from soybean (Yong et al., 2018). Additionally, other studies have indicated that planting leguminous green manure can increase the soil AP content and increase phosphorus uptake in crops, leading to improved phosphorus absorption efficiency (Pavinato et al., 2017). Our experiment supports this view, showing that, compared with monoculture, all intercropping treatments with different leguminous green manures enhanced phosphorus absorption efficiency.

### 4.2 Effects of intercropping with different leguminous cover crops on soil physical structure, nutrient content, and enzyme activity

Previous studies have shown that intercropping systems can increase nutrient contents (Wang et al., 2022). Our results confirmed that the contents of AP and SOM were higher in wheat intercropped with a leguminous green manure system than in monocropped wheat during S1, S2, S3, and S4. These findings indicate that intercropping influences nutrient dynamics by increasing soil organic carbon in the rhizosphere and changing the soil phosphorus composition. This process converts more stable phosphorus into its active form (Yang et al., 2022), thus increasing both the SOM and AP contents (Inal et al., 2007; Singh et al., 2021). Moreover, intercropping with leguminous green manure can increase soil phosphatase activity (Chamkhi et al., 2022) or activate phosphorus through the root exudates of green manure. This leads to increased phosphorus availability in the soil. In this research, we found that the soil’s AN content in the IM, IS, and IV treatments exhibited a decreasing trend at S4 compared to monocropped wheat. This decline is primarily attributed to the fact that, at this stage—when both green manure and wheat crops are at their peak growth—wheat plants have a significant demand for nitrogen, which depletes the soil’s alkaline dissolved nitrogen content (Du and Xue, 2017). Similar conclusions have been reached in other studies. Ding et al. (2021) reported that green manure application significantly increased the soil pH and effectively reduced soil acidification. In this experimental study, it was found that intercropping with leguminous green manure resulted in an increase in soil pH for wheat during the S1, S2, S3, and S4 periods compared to monocropping wheat. Among the treatments, the IS treatment exhibited the most significant increase in pH. This effect may be attributed to the greater biomass of sweet potato than of other leguminous green manures, which enhances the field water-holding capacity and improves the buffering ability of the soil against carbonates (Odlare et al., 2008), promoting a shift in soil pH toward neutrality.

Enzymes, as key factors influencing soil nutrient cycling, have activities that are closely related to plant growth (Ren et al., 2021). The experimental findings revealed that, compared to monoculture wheat, intercropping with leguminous green manure during the S1, S2, S3, and S4 periods increased the activities of URE, SUC, and ACP. This improvement may result from alterations in the physical and chemical properties of the soil due to the use of green manure. These changes facilitate the acquisition of carbon and nitrogen sources, stimulating an increase in soil microbial biomass and enzyme activity (Piotrowska and Wilczewski, 2012). A positive correlation between soil enzyme activity and SOM content has been established (Wei et al., 2015), an association confirmed in this study. Additional research indicates that intercropping leguminous green manure in rubber plantations enhances urease, catalase, polyphenol oxidase, and sucrase activities. This significantly improves soil quality and promotes rubber tree growth (Shamshuddin et al., 2004). Collectively, these results suggest that, compared with monoculture practices, intercropping systems increase soil enzyme activity and nutrient contents (Wang et al., 2014).

### 4.3 Effects of intercropping with different leguminous cover crops on the community structure and composition of soil bacteria

Planting and utilizing green manure can increase the soil nutrient content and alter microbial diversity and stability, making it a valuable agricultural strategy (Özbolat et al., 2023). This study revealed that, compared with wheat monoculture, intercropping with smooth vetch or common vetch increased both the Chao 1 index and the Shannon index. Among them, intercropping with smooth vetch significantly increased the Chao 1 and Shannon indices the most. This might be related to the fact that it had the highest soil organic matter content in each period, provided a rich source of carbon to the soil. thereby indirectly promoting the improvement of soil bacterial community diversity (Xu et al., 2023). These findings indicate that intercropping with leguminous green manure enhances soil bacterial species richness and diversity. During the maturity stage of wheat, leguminous green manure approaches the end of its life cycle, initiating decomposition. The breakdown of green manure residue improves the soil aggregate structure, loosens the soil, and creates space for microbial activity (Zhang et al., 2023c). The primary components of decomposed green manure include cellulose, lignin, and hemicellulose. Beneficial microorganisms, such as Pseudomonas and Arthrobacter, *c*an hydrolyze these components into smaller molecular compounds or monomers with enzymes such as cellulase. This process further promotes nutrient release from straw (Guo et al., 2020; Kielak et al., 2016). Consequently, the relative abundance of these beneficial bacteria and the soil nutrient content increase during decomposition (He et al., 2020). However, a sufficient nutrient supply during this process is vital for the growth of less common bacteria, which may increase bacterial community diversity.

This study revealed that Proteobacteria, Acidobacteria, Chloroflexia, and Bacteroidetes were the most abundant bacterial phyla across various intercropping systems, which is consistent with previous findings (Bai et al., 2022; Huang et al., 2022). Notably, in intercropping systems—particularly in the IC treatment—Proteobacteria levels peaked (**Figure 5A**). Many nitrogen-fixing bacteria belong to the Proteobacteria phylum (Rahav et al., 2016). Therefore, we speculate that leguminous green manure may utilize these specific bacterial communities to increase nitrogen acquisition. Acidobacteria are primarily acidophilic, thrive in acidic environments, and constitute approximately 20% of the total bacterial population (Jones et al., 2009). Research has shown an inverse correlation between Acidobacteria abundance and soil pH within a certain range (Lauber et al., 2008), with greater relative abundance in acidic soils. Our experiment confirmed this trend, as the experimental soil remained acidic throughout the study period, resulting in a relatively high relative abundance of Acidobacteria. Moreover, the dominant bacterial genera across all the treatments included *Subgroup_7*, *Sphingomonas*, *Vicinamibacteraceae*, and *KD4-96*. *Sphingomonas* is crucial for phosphate solubilization (Daniel et al., 2009). Its enrichment can improve soil nutrients and stimulate plant growth. Therefore, intercropping with various leguminous green manures can increase the abundance of beneficial bacterial phyla and genera, creating a more favorable micro-ecological environment for crops.

## 5 Conclusions

This study showed that intercropping legume green manures (IM, IC, IS, and IV) effectively improved soil physicochemical properties and enzyme activities and mitigated the risk of possible wheat yield reduction due to chemical fertilizer reduction, compared to monocropping wheat (CK), with a 30% reduction in fertilizer application, doubling the sowing density of wheat in each row (twice that of monocropping), and maintaining the same level of other field management practices. Meanwhile, the changes in soil nutrient content and enzyme activities also contributed to the changes in soil bacterial community richness and diversity, among which the IS treatment had the most significant effect on enhancing the richness and diversity of soil bacterial community, which was manifested in the growth of Chao 1 index and Shannon index. IC treatment significantly increased the relative abundance of the Proteobacteria in the soil, while IS treatment significantly increased the relative abundance of the Acidobacteria. In terms of yield performance, wheat thousand kernel weight and number of spikes were better in the IC treatment than in the IM, IS, and IV treatments, and their yields were almost equal to those of conventional wheat (CK). Overall, this study provides a scientific basis for the rational intercropping of leguminous green manure in wheat production to replace some chemical fertilizers, thus contributing to the health and sustainable development of farmland ecosystems.

## Data Availability

The original data has been uploaded to the GSA database and can be accessed via https://ngdc.cncb.ac.cn/gsa/browse/CRA032294. The project number is PRJCA049091.
